# Clutch attendance and call parameters are linked to mating success in a glassfrog with paternal care

**DOI:** 10.1093/beheco/arae078

**Published:** 2024-10-03

**Authors:** Marina Garrido-Priego, Moric Tószeghi, Francesca N Angiolani-Larrea, Anyelet Valencia-Aguilar, Lauriane Bégué, Raby Núñez, Jaime Culebras, Max Ringler, Jennifer L Stynoski, Eva Ringler

**Affiliations:** Division of Behavioural Ecology, Institute of Ecology and Evolution, University of Bern, Wohlenstrasse 50a, CH-3032 Hinterkappelen, Switzerland; Division of Behavioural Ecology, Institute of Ecology and Evolution, University of Bern, Wohlenstrasse 50a, CH-3032 Hinterkappelen, Switzerland; Division of Behavioural Ecology, Institute of Ecology and Evolution, University of Bern, Wohlenstrasse 50a, CH-3032 Hinterkappelen, Switzerland; Division of Behavioural Ecology, Institute of Ecology and Evolution, University of Bern, Wohlenstrasse 50a, CH-3032 Hinterkappelen, Switzerland; Division of Behavioural Ecology, Institute of Ecology and Evolution, University of Bern, Wohlenstrasse 50a, CH-3032 Hinterkappelen, Switzerland; Sierpefrogs, Sierpe de Osa, Puntarenas, 60503, Costa Rica; Photo Wildlife Tours, Quito, 170501, Ecuador; Fundación Cóndor Andino, Quito, 170501, Ecuador; Division of Behavioural Ecology, Institute of Ecology and Evolution, University of Bern, Wohlenstrasse 50a, CH-3032 Hinterkappelen, Switzerland; Institute of Electronic Music and Acoustics, University of Music and Performing Arts Graz, Inffeldgasse 10/III, Graz, A-8010, Austria; Instituto Clodomiro Picado, Facultad de Microbiología, Universidad de Costa Rica, San José, 11501-2060, Costa Rica; Division of Behavioural Ecology, Institute of Ecology and Evolution, University of Bern, Wohlenstrasse 50a, CH-3032 Hinterkappelen, Switzerland

**Keywords:** advertisement calls, female mate choice, *Hyalinobatrachium valerioi*, paternal care, sequential polyandry

## Abstract

Females of some species improve their reproductive success not only by being choosy and selecting males with certain traits, but also by sequentially mating with multiple males within one reproductive season. However, it is relatively unknown whether females also evaluate parental care during mate choice and, if they do, whether males actively communicate their care status to approaching females. We monitored a natural population of the glassfrog *Hyalinobatrachium valerioi*, a species with sequential polyandry and paternal care, to assess the role of parental care and advertisement calling on male mating success. Using field observations and acoustic analysis, we found that even in this species which has single-note calls, variations in call parameters allow for individual discrimination. Calling was strongly associated with mating success in *H. valerioi* males. Males with longer calls achieved the highest total mating success over the entire study period, indicating that females might have a preference for longer calls. Moreover, active calling and the presence of clutches were both linked to male mating success on a given night, although we cannot fully exclude that the link between presence of clutches and mating success is due to attractive call features alone. Call parameters differed between males when they were calling on top of their clutches, compared to sitting on the leaf directly, which might provide reliable cues about parental state to approaching females. These findings demonstrate the prominent role of acoustic communication and female choice in a species with male parental care and sequential polyandry.

## Introduction

Sexual selection is one of the key factors that have shaped the evolution of diverse mating strategies (Emlen and Oring 1977; Rosenthal 2017). In many animal species, members of the choosing sex (often females) do not mate randomly but rather exhibit preferences for certain traits of the advertising sex (Andersson 1994; Kokko et al. 2006). By being choosy, females may obtain direct or indirect benefits that ultimately will translate into increased offspring fitness (Hamilton 1990; Tregenza and Wedell 2000; Neff and Pitcher 2005; Schuett et al. 2010; Rosenthal 2017). For example, females may select males based on signals and/or cues that indicate physical and/or genetic quality such as body size (Darwin 1871; Rowland 1989; Dierkes et al. 1999; Taborsky et al. 2009), ornaments (Summers and Ord 2022), or acoustic (Zhu et al. 2017), olfactory (Parrott et al. 2007), or tactile features during courtship (Sorokowska et al. 2018).

Besides being choosy, females can also improve their reproductive success by sequentially mating with multiple males within 1 reproductive season (termed “sequential polyandry”). Thereby, females avoid the risk of selecting a single, low-quality male for all their matings and at the same time increase the genetic diversity in their offspring (Jennions and Petrie 2000; Ursprung et al. 2011). For example, in *Pseudophryne bibronii*, a terrestrial breeding frog with male parental care, more polyandrous females were found to have a higher offspring survival rate (Byrne and Keogh 2009). However, polyandry as a strategy does not necessarily preclude females from having preferences during mate choice (Byrne and Roberts 2012; Peignier et al. 2022). If females distribute their mating events across multiple partners, they might make choices based on male traits that allow for individual discrimination, such as call signatures, odors, or visual patterns (Rendall et al. 1996; Hurst 2009; Pettitt et al. 2013). Using identity information of the calls to discriminate between males (Bee et al. 2016) can help females to approach distinct males in subsequent mating events. The ability to individually discriminate between potential mating partners might be especially relevant in species with sequential polyandry (Byrne and Roberts 2012; Peignier et al. 2022). However, little is known about the role of individual call signatures for mate choice in such mating systems.

When males are the predominant caregivers, females might benefit from assessing males based on their parental performance (Clutton-Brock 1991; Burrowes 2000; Requena and Machado 2015) and distributing care across multiple males might serve as a bet-hedging strategy to decrease offspring mortality (Yasui and Garcia-Gonzalez 2016). However, to date, there is not much empirical evidence that in systems with sequential polyandry females actually evaluate potential mates based on care performance (but see, e.g., in amphibians: Pettitt et al. 2020; Valencia-Aguilar et al. 2020; fish: Matsumoto et al. 2011; arthropods: Requena and Machado 2015; mammals: Horne and Ylönen 1996, and birds: Linville et al. 1998).

In amphibians, some form of parental care is present in 56 of 76 families, and care can be provided by males, females, or both parents (Vági et al. 2019; Schulte et al. 2020; Ringler et al. 2023). Because fertilization mode is the main predictor of maternal or paternal care in this group (Furness and Capellini 2019; Vági et al. 2022) and fertilization is primarily external, we most commonly find paternal care in anurans. Although there is a large body of literature on female preferences for male traits during mate choice in many anuran species, evidence for whether male parental care is assessed by females during mate choice is scant. Furthermore, if care attracts females, males might benefit from actively communicating their care status to approaching females. In the glassfrog *Hyalinobatrachium cappellei* females prefer males with multiple clutches (Valencia-Aguilar et al. 2020), who disproportionally receive more new clutches, compared to males with fewer or no clutches. Although calling persistence did not seem to impact on males’ mating success, it is still unknown whether females exhibit a preference for certain call parameters of the single-note advertisement call of *H. capellei* males. Similarly, in golden rocket frogs (*Anomaloglossus beebei*) males who produced longer calls also provided higher-quality paternal care, and these males were preferred by females (Pettitt et al. 2020). However, it is still unclear whether, in species where males call to attract females and have parental behavior, these 2 traits are linked and play a role in males’ mating success.

In the present study, we aimed to assess the roles of parental care and calling in determining male mating success. To this end, we monitored a natural population of the glassfrog *Hyalinobatrachium valerioi*, a species with sequential polyandry and male parental care, where females approach calling males and deposit clutches on the underside of leaves that are overhanging slowly flowing streams (Savage 2002; Kubicki 2007). Clutch attendance is crucial for offspring survival and males have been observed to stay on the same oviposition site over several days and months, caring for up to 7 clutches from different females (Vockenhuber 2008; Mangold et al. 2015). While caring for clutches, males continue calling to attract more females. After hatching, tadpoles drop directly in the water, where they complete their development (Vockenhuber 2008).

We collected data on space use, behavior, and mating success (i.e. clutches) from an entire *H. valerioi* population, as well as recordings from multiple males across the entire study period, to test if (1) males show reliable interindividual call differences in the call parameters that could potentially allow for individual discrimination. We also asked (2) if certain call parameters are associated with male mating success, and (3) whether males with clutches are more attractive to females. Finally, we tested if the presence of clutches is reflected in male call parameters.

## Methods

### Study species

*Hyalinobatrachium valerioi* (Anura: Centrolenidae) is a small (snout-vent-length, herein (SVL): 19.5 to 25.0 mm), nocturnal glassfrog with a distinctive reticulated dorsal pattern and a semitransparent ventral side, occurring in rainforests ranging from Costa Rica to the Pacific Coast of Ecuador (Savage 2002; Kubicki 2007; Guayasamin et al. 2020). These arboreal frogs lay their eggs on the underside of the leaves of the vegetation overhanging streams (Savage 2002; Vockenhuber 2008). Males call to attract females to the oviposition site and amplexus can last for several hours before females lay the eggs (Vockenhuber 2008). While females leave right after oviposition, males stay caring for their clutches by hydrating, cleaning, and protecting them from predators. Male egg attendance in this species is crucial for the offspring’s survival, as otherwise they will be exposed to desiccation, fungal growth, and predation (Vockenhuber et al. 2009). Males continue calling on the same oviposition site and can attend several clutches at different developmental stages from multiple females simultaneously (Vockenhuber 2008; Mangold et al. 2015). Previous research highlighted that females mate with up to 5 different partners during a single reproductive season (Mangold et al. 2015). Once hatched, tadpoles drop into the stream, where they will further develop until metamorphosis.

Previous studies described the advertisement call of *H. valerioi* as a short high-pitched “peep” at a frequency range between 5,800 and 6,600 Hz, with a dominant frequency of 6,200 Hz, which can also rise up to 7,500 Hz (Kubicki et al. 2015; Guayasamin et al. 2020).

### Study site

We conducted our study in a lowland tropical rainforest (biome T1.1; Keith et al. 2022) along the Quebrada Negra stream in southwest Costa Rica, near the La Gamba Tropical Research Station (8.7007°N, 83.2022°W; 77 m a.s.l.). The Quebrada Negra is a smooth-flowing creek that turns into a fast-flowing stream after heavy rains. It is bordered by secondary lowland rainforest on one side and the gardens of the field station on the other side (for a detailed description of the study site and stream characteristics, see Tschelaut et al. 2008).

We conducted fieldwork from 19 September to 18 November 2021, during the breeding season of *H. valerioi* which occurs during the peak of the rainy season (Weissenhofer and Huber 2008). After a preliminary survey of the stream, we delimited a 500-m transect. To record our observations and frog locations, we first created a high-resolution map of the transect. Starting from a point of origin which we georeferenced via GPS, we set reference point markers along the transect by measuring the distance, compass direction, and inclination (for more details of the methodology used, see Ringler et al. 2014). From these reference points, we then mapped the borders of the stream using the same method as was used to set the reference points.

### Population monitoring

We performed nightly surveys (17:00 to 04:30 h) during our study period, searching acoustically and visually for males and females of *H. valerioi* and their clutches, using a ladder to reach individuals and clutches at a height of 2 to 5 m. We noted the individuals’ location, sex, and behavior, and took pictures for identification of individuals. The first image of each individual was taken on the background of 1-mm scale paper for future body measurements and subsequently was used as the reference image for that individual based on unique dorsal patterns (cf. Mangold et al. 2015). On consecutive encounters, we photographed the individuals without handling them, and compared those photos to the reference photo for identification. From reference images taken on scale paper, we also measured body size (snout-to-vent length) to the closest millimeter using the measurement tool in ImageJ (Rasband 1997–2021).

**Fig. 1.**
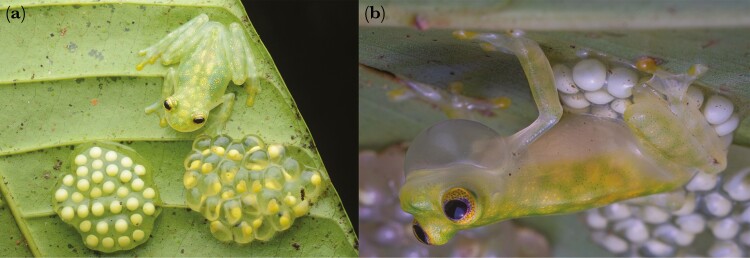
Male *Hyalinobatrachium valerioi* attending for their clutches by guarding them (a, positioned close to the clutch), and brooding (b, hydrate and clean). Credits: Raby Núñez Escalante (a), Jaime Culebras (b).

We recorded the exact location of all frogs and clutches using the mobile GIS software ArcPad 10.2 (ESRI, Redlands, CA, USA) on handheld and tablet PCs (K72H, K86H; KCOSIT Tech Ltd, Shenzhen, China) by measuring the distance and compass direction from the closest reference point. We distinguished between males and females based on observed calling activity (males) and the presence of eggs (females) when visible through their translucent body. If we were not able to unambiguously determine the sex of an individual, we scored its sex as “unknown” and updated this information if we saw the same individual calling or gravid in future encounters. For each individual, we also recorded their activity (*calling*, *in amplexus*, *courtship*), the number of clutches present near them, and if they performed parental care (*brooding, guarding, predator defense*) following the terminology of previous studies (Vockenhuber et al. 2009; Delia et al. 2013; Delia et al. 2017; Valencia-Aguilar et al. 2020; Fig. 1). We considered *brooding* when males were sitting on top of their clutch, with the ventral side of the body in contact with the eggs. *Guarding* was considered when males were near (up to 50 cm distance) or slightly touching the clutch but not directly sitting on top of it. *Predator defense* was considered when males were observed actively defending their clutches from predators. Clutches were monitored to observe male care until tadpoles hatched or until males disappeared from the oviposition site (abandonment or predation). Because there is no known multiple paternity, attendance of foreign clutches, or clutch piracy in *H. valerioi* (Mangold et al. 2015), we can confidently say that the males present at the clutches were indeed the genetic fathers. This was confirmed in another, ongoing study, using molecular parentage assignments. This was confirmed in another, ongoing study, using molecular parentage assignments (Garrido-Priego M, unpublished data).

### Recording of frog calls

Acoustic recording of frog calls was performed opportunistically during the monitoring period. As soon as we located a calling male, we approached it, using red light to minimize disturbance, and recorded it with a shotgun microphone (SSH-6, Zoom Corporation, Tokyo, Japan) attached to a digital recorder (H5, Zoom Corporation) with a sampling rate of 44.1 kHz and 16-bit resolution. Whenever possible, we recorded males from a 50-cm distance (range: 50 to 200 cm) and up to 5-m height using a telescoping pole to which we attached the recorder and microphone. We first recorded males for 10 min to allow for an analysis of larger bout structures, and on subsequent encounters for 3 min or for at least 30 undisturbed calls. Immediately afterwards, we measured temperature to the closest 0.1 °C using a thermometer (HY-10 TH, Voltcraft, Hirschau, Germany). All frogs were recorded before any handling took place as described in *Population monitoring*.

### Acoustic analysis

**Fig. 2.**
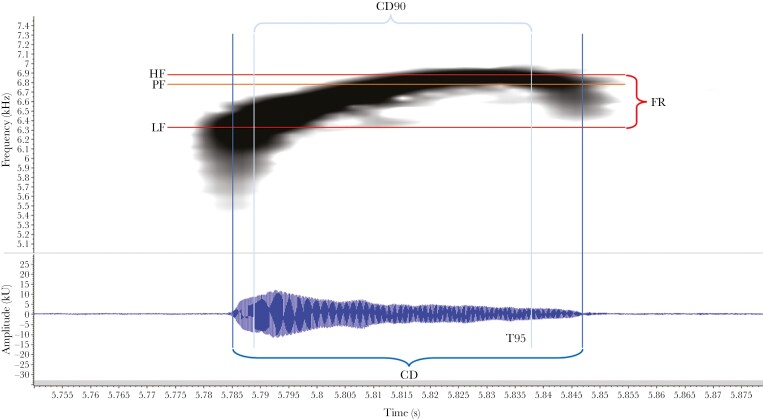
Representation of the spectrogram (top) and the waveform (bottom) of an advertisement call of *Hyalinobatrachium valerioi*. CD: total duration of the call, in dark blue. CD90: call duration when the initial and final 5% of the sound energy is skipped, leaving the central 90% of the sound energy. T95: the relative time within a call when 95% of the sound energy has occurred. HF: high frequency, LF: low frequency, FR: frequency range. PF: peak frequency, the frequency at which most sound energy is stored.

We analyzed the recordings with the acoustic analysis software Raven Pro 1.6 (K. Lisa Yang Center for Conservation Bioacoustics at the Cornell Lab of Ornithology, 2022), applying a band-limited energy detector (settings: frequency: 5,000 to 10,000 Hz; duration: 0.1 to 1 s; minimum separation: 0.2 s; minimum occupancy: 60%; signal-to-noise ratio threshold: 21%; noise block size: 1 s; noise hop size: 0.5 s; noise percentile: 20%) to find and select frog calls in the recordings. From the verified detections we extracted the following temporal parameters: duration of the call (CD in ms); call duration when the initial and final 5% of the sound energy was skipped, leaving the central 90% of the sound energy (CD90 in ms) which is less sensitive to outliers or noise; interval between the end of a call and the beginning of the next call (ICI in s); the relative time within a call when 95% of the sound energy has occurred (T95 in %) (for a graphical representation, see Fig. 2). We also extracted the following spectral parameters: peak frequency (PF in Hz), the lowest frequency (LF in Hz), highest frequency (HF in Hz), and frequency range (FR in Hz) calculated as the difference between HF and LF. Temporal parameters were analyzed using waveform and spectrogram views with fast-Fourier transformation (FFT window size: 64, Blackman window), whereas spectral parameters were analyzed in the spectrogram view only with an FFT window size of 1,024 (time grid overlap: 90%, hop size: 96 samples). In some cases, call duration as measured by the automatic detector was wrong, apparently due to strong reverberation, so the measurement was adjusted manually. Descriptive statistics of each call parameter were calculated across all calls (>15,000 calls from 279 recordings of 26 males) using separate linear mixed-effect models with the function *lmer* in the package *lme4* (Bates et al. 2003). We used “male ID,” “recording ID,” and “parental behavior” as random effects and each call parameter as fixed effects.

### Individuality of the advertisement call

To assess between-individual variation in the advertisement calls, we randomly selected 3 recordings from each male, as this was the minimum shared number of recordings per male. We excluded recordings from males calling on clutches in this analysis after we identified that males occasionally call while brooding, and these calls differ from those produced when males are sitting on the leaf directly (see *Results* section). We only included recordings with 30 or more calls of which we omitted the first 5 calls to account for potential disturbances at the onset of recording. Thus, we analyzed 78 recordings from 26 males (3 recordings per male) with at least 25 calls per recording (1,950 calls in total).

Males do not call continuously but rather take breaks from calling at irregular intervals. The first call of a new call sequence is significantly shorter than subsequent calls (0.055 s vs. 0.065 s; Welch’s *t*-test: *t* = 11, degrees of freedom [df] = 196, *P* < 0.001) and has lower amplitude (8,194 Hz vs. 11,167 Hz; Welch’s *t*-test: *t* = 12, df = 198, *P *< 0.001). If a recording included several call sequences, we therefore excluded the first call of each sequence. As the number of calls per recording was quite variable (average: 55 ± 42).

To identify call properties that would allow for individual discrimination, we calculated the coefficient of variation (*CV* = $\frac{SD}{Mean}*100$) for every call parameter and individual. We then compared the variation within (*CVw*) and between (*CVb*) individuals (Gerhardt 1991; Gasser et al. 2009; Wei et al. 2019). For every parameter we calculated the *CVw* as the average *CV* across all 3 recordings from each frog, and the *CVb* from the mean and standard deviation across all 1,950 calls.

We also determined Beecher’s information capacity (*Hs*) to create a comprehensive measurement of individuality information content by integrating all call parameters into a single estimate (Beecher 1989). First, we performed a principal component analysis (PCA) on the 8 call parameters (principal components, herein PCs), using the *calcPCA* function in the *IDmeasurer* package (Linhart et al. 2019) to get uncorrelated variables. We used all 8 PCs to calculate the total *Hs* using the *calcHS* function in the *IDmeasurer* package (Linhart et al. 2019). Following Tumulty et al. (2018), we then used a more conservative approach in which we only used the 3 PCs that had eigenvalues greater than 1. We were further interested in whether the information content of the calls is sufficient to differentiate between individuals in a biologically relevant context, i.e. in their natural environment. As *Hs* states information capacity in bit, 2^*Hs*^ gives the maximum group size in which an individual can be identified based on the assessed parameters (Beecher 1989). Using our field observations, we assessed the number of neighbors of every calling male within a range of 15 m and compared this number to the maximal discriminable group size for *H. valerioi* based on the information capacity of the call.

To calculate the probability with which a call can be assigned to the correct male, we used a linear discriminant analysis (LDA) based on the previous 8 PCs for each male.

### Link between call parameters and male total mating success

We investigated whether specific call parameters are associated with the total mating success of an individual across the study period. We fit a generalized linear model (GLM) with a Poisson distribution, using the function *glm*. We used “total number of clutches” to measure the total mating success of each male across the entire study period as the response variables and included “body size” and the individual averages of “call duration,” “inter-call interval,” “95% time,” “peak frequency,” and “frequency range” as fixed effects.

### The role of calling and clutch attendance in males’ mating success

To analyze whether the presence or absence of clutches (i.e. recent mating success) at the male’s location impacts a male’s mating success on a given night, we fit a generalized linear mixed-effects model (GLMM) with a binomial distribution, using the function *glmer* in the *lme4* package. We considered mating success on a given night when a male was seen in amplexus and a clutch was laid by the female. If the amplexus did not result in a clutch, we did not consider this event as mating success. For this analysis, we used information from 48 males monitored through the entire study period. We used “male mating success” in a given night (yes = 1, no = 0) as the response variable, and included both “presence/absence of clutches” (yes = 1, no = 0) at that site on the same night and whether the focal male was “calling” (yes = 1, no = 0) as fixed effects. We used “male ID” as a random effect to control for multiple observations per male. As body size was not repeatedly assessed for every encounter, we did not include this variable in this model.

### Do call parameters reflect parental behavior?

We investigated whether the presence of clutches is reflected in males’ call parameters. The field observation that males not only call next to their clutches but also when sitting on top of them motivated us to test if calls produced in different contexts differ in their spectral or temporal parameters. Thus, we considered 3 “calling conditions”: calls produced in absence of clutches (no clutches), calls produced sitting next to clutches (guarding), and calls produced on top of clutches (brooding). We used all recordings of males (*N* = 279), resulting in a total of 15,278 calls, for this analysis.

To check whether parental behavior is associated with any of the call parameters, we fit a linear mixed-effect model for each call parameter using the function *lmer* from the *lme4* package. We used the call parameters as response variables and “calling condition” (categorical variable with 3 levels: no clutches, brooding, or guarding), temperature (continuous variable), and if there was rainfall during the recording (binary variable: yes/no) as fixed effects. We included “male ID” as a random effect to control for multiple observations per male. As temporal parameters have previously been shown to be affected by ambient temperature (Lingnau and Bastos 2007; Ziegler et al. 2016) and calling behavior in *H. valerioi* is positively correlated with the amount of daily rainfall (Vockenhuber 2008), we also included “temperature” and “rainfall during the recording” into the model. As body size was not repeatedly assessed for every encounter, we did not include this variable in the model. Statistical outcomes were obtained using the function anova. *P*-values were corrected using the weighted Bonferroni–Holm method to account for multiple testing across the 8 separate models. Finally, we performed pairwise post hoc comparisons between “calling conditions” (no clutches/brooding/guarding) for only those call parameters that showed significant results using Estimated Marginal Means with Tukey contrasts using the function *emmeans* from the *emmeans* package (Lenth 2024).

All analyses were carried out in R version 4.1.2 (R Core Team 2024), using the integrated development environment RStudio version 2023.06.1 (RStudio Team 2024). We report and interpret the results following the recommendations by Muff et al. (2022); *P* > 0.1 no evidence, 0.1 < *P* < 0.05 weak evidence, 0.05 < *P* < 0.01 moderate evidence, 0.01 < *P* < 0.001 strong evidence, *P* < 0.001 very strong evidence.

## Results

### Population monitoring

We monitored a total of 102 *H. valerioi* (48 males, 54 females), obtaining a total of 1,199 individual observations (1,053 males, 146 females). Males were found during 16.04 ± 13.56 (mean ± SD here and throughout text) nights on average. Successful males had on average 4.7 ± 2.37 clutches over the entire study period and took care of a maximum of 6 clutches at the same time. Of these 48 males, we only used those males (*N* = 26) for the acoustic analysis of call parameters where we obtained 3 recordings with at least 25 calls each recording.

### Descriptive statistics and individuality of advertisement calls

The advertisement call of *H. valerioi* at our field site consists of a single, very short high-pitched note with a mean duration of 63.9 ms (range: 14 to 94 ms) and mean peak frequency of 6,724 Hz (range: 5,728 to 7,924 Hz), which is repeated every 4.04 s (range: 0.32 to 18.86 s) on average (values are model estimates; see a more detailed description of the call in Table 1). All parameters were more variable between than within individuals, as revealed by the *CVb/CVw* ratios which were all larger than 1 (Table 2). Given that all call parameters could potentially contribute to the individual acoustic identity, they were all incorporated in the PCA, and subsequently for the estimation of the information capacity.

**Table 1.** Descriptive statistics of the temporal and spectral parameters for all males (*N* = 26) across 279 recordings.

Parameters	Mean	Range	Within-male (*CVw *± SD [range])	Between-male (*CVb*)	Ratio of *CVb/CVw*
Temporal parameters					
CD—call duration (s)	0.064	0.014 to 0.094	8.08 ± 4.01 [2.16 to 17.48]	13.67	1.69
CD90—90% call duration (s)	0.046	0.011 to 0.072	9.47 ± 5.15 [1.02 to 22.50]	16.61	1.75
ICI—inter-call interval (s)	4.04	0.32 to 18.86	18.3 ± 14.2 [1.93 to 63.50]	36.21	1.98
T95—relative time 95% (%)	78.9	30.7 to 59	5.14 ± 4.06 [1.07 to 16.71]	9.97	1.94
Spectral parameters					
PF—peak frequency (Hz)	6,724.3	5,728 to 7,924	1.37 ± 0.67 [0.37 to 2.85]	3.58	2.62
LF—low frequency (Hz)	6,439.9	5,556 to 7,321	1.34 ± 0.91 [0.26 to 4.35]	2.75	2.06
HF—high frequency (Hz)	7,153.1	6,288 to 8,398	1.59 ± 1.30 [0.15 to 5.54]	5.22	3.29
FR—frequency range (Hz)	718.5	215 to 2,110	12.95 ± 8.67 [1.16 to 34.04]	43.65	3.37

Mean was calculated using linear mixed-effect models with recording ID and male ID as random effects (to account for different numbers of calls per recording, and recordings per male, respectively) and every call parameter as a response variable. Range is calculated across all calls (*N* = 15,278 calls). *CV* for every parameter at the within-male (*CVw*) and between-male (*CVb*) levels.

**Table 2.** PCA of the 8 call parameters.

	PC1	PC2	PC3	PC4	PC5	PC6	PC7	PC8
CD	8.7	21.6	16.4	2.6	12.6	0.4	37.6	0
CD90	14.8	24.3	0.1	8	8	1.9	42.9	0
ICI	0	8.6	21.5	5.9	63.8	0.2	0	0
T95	4.2	1	30.9	44.6	0.8	0.2	18.3	0
PF	10.5	21.7	10.7	1.9	0	54.4	0.7	0
LF	11.8	20.1	7.9	9.1	1.6	37.5	0.5	11.5
HF	29.7	2.1	2.0	6.3	3.9	4.5	0.1	51.4
FR	20.3	0.7	10.4	21.6	9.2	0.9	0	37.1
Eigenvalues	2.8	1.8	1.45	0.91	0.75	0.26	0.02	0
Variance (%)	35.11	22.49	18.14	11.4	9.39	3.22	0.25	0
Cumulative variance (%)	35.1	57.6	75.8	87.2	86.5	99.8	100	100

Our population monitoring revealed that calling males were never surrounded by more than 5 other males within a range of 15 m. Based on the 10 PCs (Table 2) used to calculate Beecher’s *Hs*, the total information capacity of the call was 3.01 bits, meaning that the call properties allow identifying an individual in a group of up to 8 (2^3.01^ = 8.05) calling males. The more conservative estimate, using only the 3 PCs that had eigenvalues greater than 1 (which together explain 75.8% of the total variance; Table 2) yielded a total information capacity of 2.37 bits, corresponding to a maximum group size of 5 (2^2.37^ = 5.17) uniquely calling individuals. In the 2D space created by LD1 and LD2 from our LDA, no individual can be fully separated from the others based on the call parameters. However, the calls of most males are strongly concentrated and compactly arranged around the “average call” of the respective individuals. Moreover, 51.9 ± 24.5% of the calls could be assigned to the correct male, compared to the 3.8% (1/26) by chance.

### Link between call parameters and male mating success

**Fig. 3.**
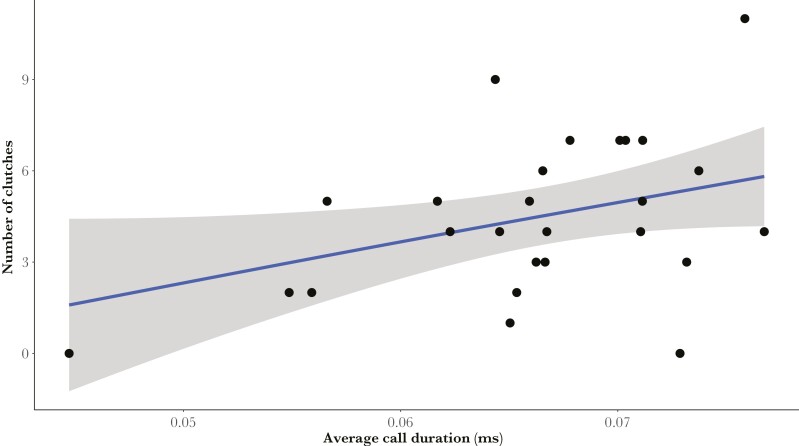
Positive correlation between the total number of clutches at the end of the study period (total mating success) and call duration in *H. valerioi* males (*R*^2^ = 0.05).

We found strong evidence that “call duration” was positively linked to total mating success of males (GLM: β = 73.02, *Z* = 2.95, *P* = 0.003; Table 3; Fig. 3), as males with longer calls had accumulated significantly more clutches at the end of the sampling period. There was no evidence that any of the other parameters had a significant impact on the total number of clutches that each male obtained throughout the entire study period (Table 3).

**Table 3.** Summary of the results obtained from the fitted models.

Model	Fixed effects	*N*	Estimate	SE	*Z/t* value	*P*-value
1. Total number of clutches	Intercept	26	−8.82	8.11	−1.09	0.27
	Body size		−0.07	0.13	−0.54	0.58
	CD		73.02	24.71	2.95	0.0031
	ICI		0.12	0.18	0.67	0.5
	T95		6.18	4.18	1.48	0.14
	PF		0.00	0.00	0.29	0.77
	FR		0.00	4.18	1.48	0.14
2. Male mating success	Intercept	48	−5.2894	0.73	−7.205	<0.001
	Calling		3.5396	0.72	4.917	<0.001
	Presence/absence of clutches		0.5671	0.23	2.448	0.014

1. Result of the GLM fitted to test for an association between call parameters and mating success. 2. Result of the GLMM to test for the role of “calling” (yes/no) and “clutch attendance” (yes/no) in “male mating success” (yes/no).

### The role of calling and clutch attendance in males’ mating success

We found very strong evidence that calling increased the likelihood to successfully mate in a given night (GLMM: β = 3.54, *Z* = 4.92, *P* < 0.001; Table 3). We also found strong evidence that the presence of clutches at the calling site of a male increased his likelihood of obtaining an additional new clutch in a given night (GLMM: β = 0.57, *Z* = 2.45, *P* = 0.014; Table 3).

### Do call parameters reflect parental behavior?

We found very strong evidence that call duration (Chi-sq = 23.57, df = 2, *P* = <0.001), and inter-call interval (Chi-sq = 17.99, df = 2, *P* = <0.001) differed across calling conditions (Table 4). Furthermore, we found very strong evidence that temperature was also associated to call duration (Chi-sq = 20.04, df = 1, *P* = <0.001).

**Table 4.** Result of the LMER models to test for the association of “calling condition,” “rainfall,” and “temperature” on temporal and spectral call parameters.

Model	Fixed effects	Chi-sq	df	*P-*value (Bonferroni)	
CD	Calling condition	23.57	2.00	<0.001	
	Rainfall	2.95	1.00	1.00	
	Temperature	20.04	1.00	<0.001	
CD90	Calling condition	8.62	2.00	0.312	
	Rainfall	0.17	1.00	1.000	
	Temperature	9.53	1.00	0.048	
ICI	Calling condition	17.99	2.00	<0.001	
	Rainfall	8.25	1.00	0.096	
	Temperature	1.16	1.00	1.000	
T95	Calling condition	8.40	2.00	0.360	
	Rainfall	6.20	1.00	0.312	
	Temperature	3.79	1.00	1.000	
PF	Calling condition	1.93	2.00	1.000	
	Rainfall	0.28	1.00	1.000	
	Temperature	0.65	1.00	1.000	
LF	Calling condition	0.17	2.00	1.000	
	Rainfall	3.48	1.00	1.000	
	Temperature	0.10	1.00	1.000	
HF	Calling condition	9.46	2.00	0.216	
	Rainfall	3.51	1.00	1.000	
	Temperature	0.01	1.00	1.000	
FR	Calling condition	4.25	2.00	1.000	
	Rainfall	0.95	1.00	1.000	
	Temperature	0.13	1.00	1.000	
Model	Predictor	Pairwise comparison	Estimate ± SE	*t*-ratio	*P*-value
CD	Calling condition	Brooding vs. Guarding	−0.005 ± 0.001	−4.740	<0.001
		Brooding vs. No clutches	−0.003 ± 0.001	−2.740	0.0179
		Guarding vs. No clutches	0.002 ± 0.001	2.350	0.051
ICI	Calling condition	Brooding vs. Guarding	0.719 ± 0.171	4.230	<0.001
		Brooding vs. No clutches	0.532 ± 0.179	3.000	0.008
		Guarding vs. No clutches	−0.187 ± 0.125	−1.500	0.292

*P*-values in bold are significant at the 0.05 level after adjusting for multiple testing using the *weighted Holm–Bonferroni method*. Pairwise comparison of the 3 levels of the variable “calling condition”: brooding, guarding, and no clutches for call duration (CD) and inter-call interval (ICI) in a post hoc test with Tukey adjustment.

**Fig. 4.**
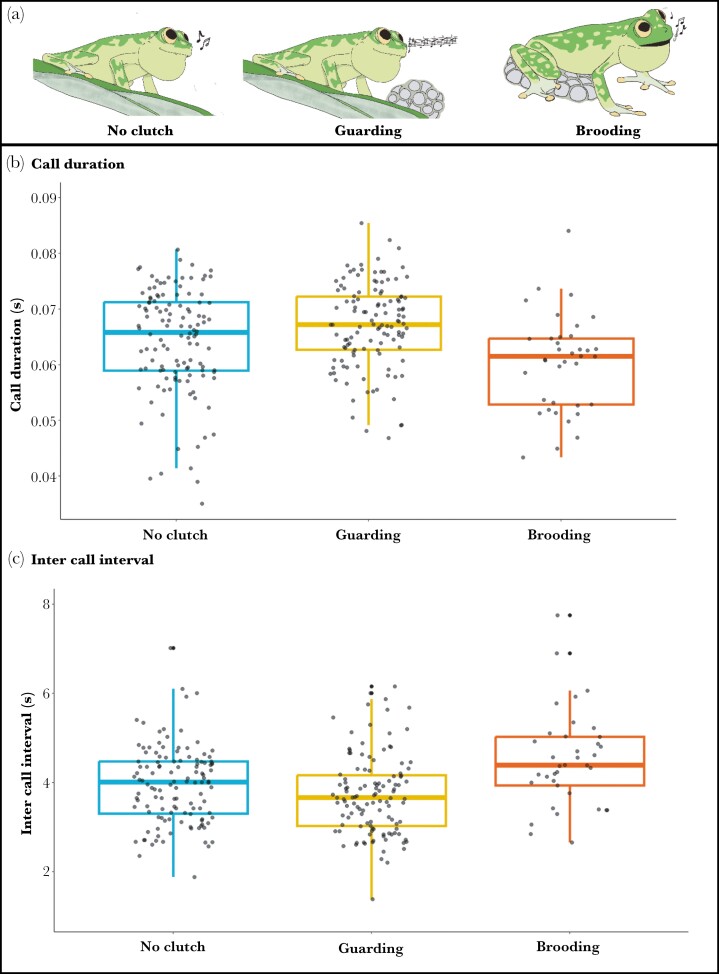
(a) Illustration of the 3 types of calls compared in this analysis: male with no clutches, guarding, and brooding. (b) Call duration (CD) was shorter in brooding calls compared to guarding (*P* < 0.0001) or no-clutch (*P* = 0.0179) calls. (c) Inter-call interval (ICI) was longer in brooding calls compared to guarding (*P* < 0.0001) and no-clutch (*P* = 0.007) calls. CD and ICI were no different between attending and no-clutch calls (*P* > 0.05).

We found very strong evidence that the mean call duration from brooding calls was shorter than from guarding calls (β = −0.0052, standard error [SE] = 0.001, *P* < 0.001; Fig. 4b; Table 4) or calls from males with no clutches (β = −0.0032, SE = −0.001, *P* = 0.0179; Fig. 4b; Table 4). We also found evidence that the mean inter-call intervals from brooding calls were longer than from guarding calls (β = 0.719, SE = 0.171, *P* < 0.001; Fig. 4c; Table 4) or calls from males with no clutches (β = 0.532, SE = 0.179, *P* = 0.008; Fig. 4c; Table 4). We found weak evidence that call duration was longer in guarding calls compared to calls from males with no clutches (β = 0.002, SE = 0.001, *P* = 0.051; Table 4), but no evidence that inter-call interval was different between those 2 calling conditions (β = −0.187, SE = 0.125, *P* = 0.291; Table 4).

## Discussion

In this study, we investigated possible factors shaping male mating success in a glassfrog with male parental care. The acoustic analysis revealed that calls of *H. valerioi* males are individually distinct and allow for discrimination of individuals. Males that were already attending clutches were more likely to have mating success on a given night than males without clutches, and males that produced on average longer calls had higher mating success across the entire study period, than those with shorter calls. However, the calls of males produced when sitting on top of their clutches were shorter than those of other males.

### Individuality of advertisement calls

The *CVb/CVw* ratios of all analyzed acoustic parameters were larger than 1, indicating that calls are more variable between than within individuals. Overall, the temporal call parameters varied more than the spectral call parameters, yet all analyzed call parameters contributed to individual acoustic identity, with the spectral parameters being more individually distinctive (higher *CVb/CVw* ratio). The *CVb/CVw* ratios obtained in our study were in the same range as the ratios found in a previous study, in which male olive frogs (*Babina adenopleura*) successfully distinguished neighbors from nonfamiliar individuals in playback experiments (see, e.g., Chuang et al. 2017). An LDA further revealed that calls of male *H. valerioi* could be assigned to the correct male at 13 times the probability expected by chance (51.9% vs. 3.9%), which further corroborates the existence of between-individual differences in advertisement calls. These findings suggest that not only male, but also female *H. valerioi* could use this information to discriminate between different males based on their individual call signatures. In natural settings, a female can rarely, probably never, assess all of the males of a given population at the same time. Therefore, to obtain a more biologically relevant view on individual acoustic discrimination in natural situations, we looked at the variation in the acoustic parameters of subgroups (Bee and Gerhardt 2001; Gasser et al. 2009; Bosch et al. 2017; Tumulty et al. 2021). Based on our population monitoring data, we determined that a calling male was never adjacent to more than 5 other conspecifics. Beecher’s *Hs* of the *H. valerioi* calls revealed an information capacity of 3.01 bits, which would allow for effective discrimination of 8 individuals, and thus is probably sufficient for individual *H. valerioi* to distinguish between calling males in a natural calling aggregation. So far, vocal individuality has mostly been studied in birds (Carlson et al. 2020), most likely because their highly complex and variable calls are more likely to allow for between-individual differences. Previous studies in anurans have shown vocal individuality in several species that exhibit pulsed and multi-note calls (e.g. Bee and Gerhardt 2001; Bee et al. 2001; Gasser et al. 2009; Bosch et al. 2017). Here we show that even calls with a very simple structure, like those of *H. valerioi,* can contain sufficient between-individual variation. However, whether individuals, in this case females, could actually use this information to discriminate between distinct males, remains to be tested.

### Link between call parameters and male mating success

We found that several call parameters were strongly associated with mating success in *H. valerioi* males. Males that produced on average longer calls had higher mating success across the study period, compared to males with shorter calls, suggesting that female *H. valerioi* might prefer males with long calls. Female preference for specific call parameters such as call duration or peak frequency has been demonstrated in numerous anuran species (Pettitt et al. 2020; Cheng et al. 2022; Peignier et al. 2022). In *A. beebei*, females preferred males with longer calls, which were also the ones that had better parental abilities (Pettitt et al. 2020). Longer calls require more energy, and thus represent an honest signal for male calling effort (Forsman and Hagman 2006). Unfortunately, we have limited information about the relationship between male physical quality, calling effort, and parental performance. Further studies are needed to investigate the causal relationship between these factors and their impact on individual fitness.

### The role of calling and clutch attendance on male mating success

We found very strong evidence that calling was associated with the likelihood of obtaining a new clutch on a given night. On one hand, males that are calling are likely more conspicuous to females, raising the probability of being detected and subsequentially selected (Meuche et al. 2013; Mangold et al. 2015). On the other hand, calling is energetically expensive and may represent an honest signal for physical quality (see section above).

We also found that the presence of clutches increased a male’s likelihood of successfully mating on a given night. Similar findings were reported in another glassfrog species *H. cappellei* (Valencia-Aguilar et al. 2020), demonstrating that previous mating success is attractive to females. This phenomenon can also be observed in other taxa, such as fishes and arthropods in which females preferentially mate with males that are attending clutches (Ridley and Rechten 1981; Matsumoto et al. 2011; Requena and Machado 2015; Ohba et al. 2018). All these findings support the “parental care hypothesis” proposed by Byrne and Roberts (2012) according to which paternal care itself is an attractive trait which is evaluated by females during mate choice. Given that multiple paternity within clutches does not occur in this species (Mangold et al. 2015), the presence of clutches on a leaf with an *H. valerioi* male serves as an honest indicator for previous mating success. Previous mating success could even allow for females to employ a copying strategy during mate choice, by relying on the assessment of a given male by previous females as a suitable mating partner (Sirot 2001).

Males with clutches are more likely to remain at a given location (oviposition site), thereby reducing the likelihood of clutch desertion by the male after mating. In fact, male presence at the oviposition site and active care have been demonstrated to significantly enhance offspring survival in several species of glassfrogs from the genus *Hyalinobatrachium* and *Centrolene* (Vockenhuber et al. 2009; Delia et al. 2013, 2017). Therefore, male parental performance is a key factor for female fitness, and evaluation and selection of males based on their parental performance will provide a strong selective advantage for females. However, it is still unknown if and to what degree males vary in the quality of provided care. Furthermore, the methodological setup of our study cannot clearly disentangle the effect of clutch presence per se from other traits that make a male attractive in the first place, such as call parameters (see section above). If males with long calls are attractive, then subsequent mating success could be due to call parameters alone, and the association between mating success and clutch attendance cloud be a mere byproduct of the effect of attractive calls. Future research is needed to determine if *H. valerioi* males vary in their parental performance and if females incorporate this information in their mating decisions.

### Do call parameters reflect parental behavior?

Being poikilotherms, the body temperature of frogs directly depends on the ambient temperature, with increasing temperature resulting in an increased metabolic rate. This metabolic change also directly affects calling and leads to increased call rates and higher call frequencies on the one hand, and shorter calls and shorter intervals between notes and calls on the other hand, when temperature increases (Gillooly et al. 2001; Narins and Feng 2006). This effect has been shown in many frog species e.g. *Hylodes heyeri* (Lingnau and Bastos 2010), *Hypsiboas pulchellus* (Ziegler et al. 2016), and also in our study in *H. valerioi* we found strong evidence that call duration decreased with increasing temperature, while other parameters were not affected. However, as in our study we did not perform a time series analysis of reproductive success, we cannot assess to what extend changes in temperature might differentially affect the reproductive success of individual males. As calling generally is energetically costly (Prestwich 1994; McLister 2021), the increased energy expenditure coming from higher calling rates at higher temperatures might impose energetic constraint on frogs under the current climatic change (cf. Schlippe et al. 2022).

Calls emitted by brooding males were shorter and the intervals between the calls were longer (i.e. lower call rate) than those from males that were sitting next to clutches or males without clutches. As parental care and calling are both energetically costly, males might compensate the increased parental effort by reducing the amount of energy invested in calling (Gerhardt and Huber 2002; Köhler et al. 2017; Escalona Sulbarán et al. 2019). A lower call rate could also reflect certain constraints on male calling behavior that are imposed by different calling substrates (clutch vs. leaf). Unfortunately, our experimental design cannot confirm whether the differences in call parameters between brooding and nonbrooding males are the result of males actively modifying their calls or the impact of the calling substrate. Theoretically, these distinct calls by brooding males could be an honest signal to females for active parental care by males, irrespective of whether the males intentionally transmit this information or if this is just passed as a byproduct of alterations by the calling substrate. However, we did not find evidence of differences in call parameters between males with varying numbers of clutches, and the acoustic analysis revealed that males with longer calls actually had a higher total mating success (see section *Link between call parameters and male mating success*). Nevertheless, we cannot exclude the possibility that other call parameters that we did not measure (e.g. call shape) indicate that a male is brooding, and that females exhibit a preference for those parameters. Playback experiments, in which females are presented with synthetic calls that mimic brooding and nonbrooding males are needed to reveal if females can detect and use differences in call parameters of brooding and nonbrooding males in their mating decisions.

## Conclusion

Our study demonstrates that even calls with a very simple structure can contain sufficient between-individual variation in temporal and spectral parameters to allow for individual discrimination. The ability to discriminate between potential mating partners might be especially relevant to females in species with sequential polyandry. Calling was strongly associated with mating success in *H. valerioi* males. Calling makes males more conspicuous, but because calling is also energetically expensive it may represent an honest signal for physical quality. Unfortunately, we have limited information regarding the ways in which male physical quality, calling effort, and parental performance are linked. In species with paternal care, females might benefit from assessing male parental performance when choosing a mating partner. However, the higher mating success of males that were attending clutches could also be the result of female copying other females’ mating decisions, or a byproduct of other traits (such as call parameters) preferred by females that indicate male quality. The finding that calls emitted by brooding males were shorter than calls by nonbrooding males may reflect the energy trade-off between parental care and acoustic signaling. However, males with longer calls obtained the highest mating success over the entire study period, suggesting that females pay attention to high-cost signals more so than parental state. These findings demonstrate the prominent role of acoustic communication and female choice in a species with male parental care and sequential polyandry.

## Data Availability

Analyses reported in this article can be reproduced using the data provided by Garrido-Priego et al. (2024).
